# Ocular Antibiotic Utilisation across Aotearoa/New Zealand

**DOI:** 10.3390/antibiotics12061007

**Published:** 2023-06-04

**Authors:** Isabella M. Y. Cheung, Simon Horsburgh, Mohammed Ziaei, Akilesh Gokul

**Affiliations:** 1 Department of Ophthalmology, University of Auckland, Auckland 1023, New Zealand; 2 Department of Preventive and Social Medicine, University of Otago, Dunedin 9016, New Zealand

**Keywords:** drug utilisation, anti-bacterial agents, eye

## Abstract

Ocular antibiotics are integral to the prevention and treatment of bacterial ocular infections. This study aimed to describe their utilisation across New Zealand according to patient and healthcare factors. Every subsidy-eligible community dispensing of ocular chloramphenicol, fusidic acid and ciprofloxacin in New Zealand, between 2010 and 2019, was included in this analysis. Number of dispensings/1000 population/year was quantified, stratified by patient age and urban/non-urban health districts. Dispensing rates by ethnicity were determined and were age adjusted. The proportion of dispensings by socioeconomic deprivation quintile was also determined. Chloramphenicol was the most commonly dispensed antibiotic; however, its utilisation decreased over time. Ciprofloxacin use was higher in children, while chloramphenicol use was higher in older patients. Ciprofloxacin usage was higher among Māori and Pasifika ethnicities, while fusidic acid use was lower. Chloramphenicol usage was higher among Pasifika. Antibiotic utilisation was higher in urban health districts, and in the most deprived quintile; both were most marked with ciprofloxacin. The utilisation of publicly funded ocular antibiotics across New Zealand varied between patient subgroups. These findings will help improve the prevention, management and outcomes of bacterial ocular infections, and support wider initiatives in antibiotic stewardship and medicine access equity.

## 1. Introduction

Ocular antibiotics are integral to the prevention and treatment of bacterial ocular infections. In New Zealand (NZ), chloramphenicol, fusidic acid and ciprofloxacin are the most commonly utilised therapies [1]. Chloramphenicol is used prophylactically following cataract surgery to prevent endophthalmitis, and empirically in relatively common conditions such as presumed infective conjunctivitis [2,3]. Fusidic acid is employed in staphylococcal keratitis, and in external conditions such as infective blepharitis [4,5]. Ciprofloxacin is generally reserved for the treatment of bacterial keratitis [6].

Little is known about ocular antibiotic utilisation across NZ. The utilisation of some ocular antibiotics has been increasing in other countries such as Spain and China [7,8]. Notably, rising levels of ciprofloxacin resistance in ocular isolates have been detected in NZ [9]. It also remains unclear whether the utilisation of ocular antibiotics in NZ is equitable. Systemic antimicrobial consumption may be lower among Māori (the indigenous peoples of NZ); however, the incidence of all-cause infectious disease may be higher among Māori and Pasifika (the indigenous peoples of the South Pacific islands) [10,11]. Systemic antimicrobial use is also lower in rural NZ, particularly among rural Māori, possibly resulting from barriers to healthcare access [11]. In contrast, higher systemic antimicrobial consumption in NZ has been linked to higher socioeconomic deprivation in some studies, likely due to a link between deprivation and higher infection rates [10,12]. This study aimed to describe the utilisation of publicly funded ocular antibiotics across NZ over a decade, stratified by patient and healthcare factors. Broadly speaking, this study found that utilisation varied between population subgroups.

## 2. Results

**Figure 1:**
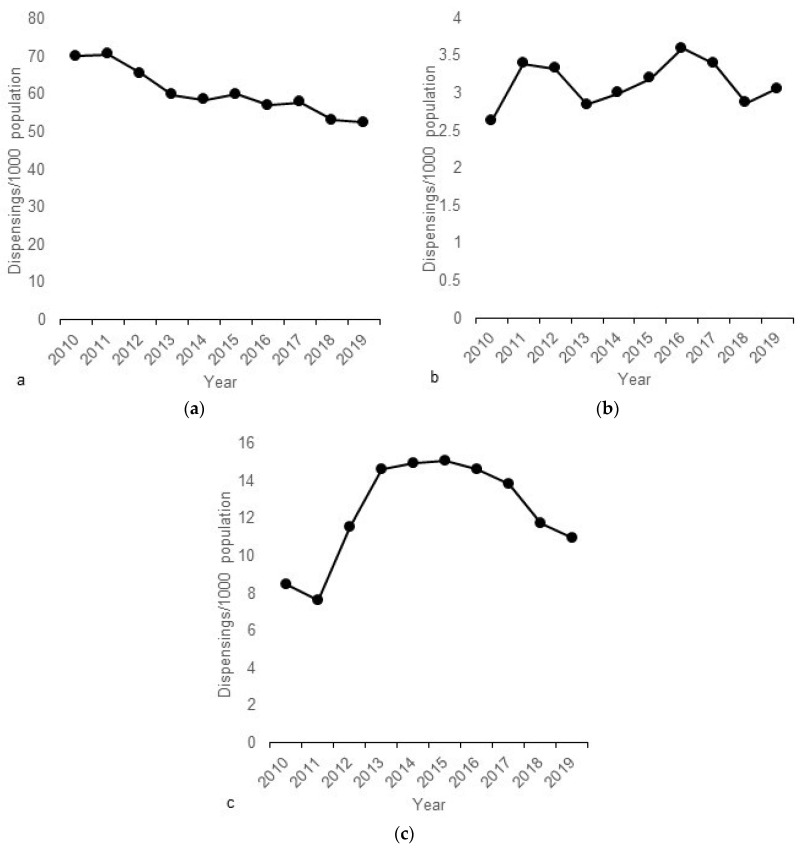
Number of dispensings per 1000 population by year: (**a**) chloramphenicol; (**b**) ciprofloxacin; (**c**) fusidic acid. Note the varying scale of the *y*-axis between graphs.

There were 2,682,407 subsidy-eligible dispensings of chloramphenicol, 551,754 of fusidic acid and 139,313 of ciprofloxacin over the 10-year study period (Table 1). Chloramphenicol dispensings declined over the study period (Figure 1a), while ciprofloxacin dispensing remained relatively constant (Figure 1b). Fusidic acid dispensings rose initially between 2010 and 2013, but subsequently declined (Figure 1c).

Ciprofloxacin dispensings in children were approximately double that in older individuals (mean ± SD of 5.8 ± 0.60 and 3.0 ± 0.32/1000 people/year, respectively, *p* < 0.001). In contrast, chloramphenicol dispensings were significantly higher in older patients than in children (115 ± 16.3 and 98 ± 9.2/1000 people/year, respectively, *p* = 0.01). Fusidic acid dispensings in children and older individuals were not significantly different (23.5 ± 7.2 and 23.0 ± 3.0/1000 people/year, respectively, *p* = 0.84).

Ciprofloxacin dispensings were significantly higher among Māori and Pasifika, compared with non-Māori/non-Pasifika (both *p* ≤ 0.05, Table 2). For chloramphenicol and fusidic acid, there were no significant differences among Māori and Pasifika, compared with non-Māori/non-Pasifika (all *p* ≥ 0.05). However, chloramphenicol dispensings were significantly higher among Pasifika when compared with Māori (*p* = 0.006).

A sub-analysis by age (specifically 0–14 years and over 65 years) was performed for chloramphenicol and fusidic acid. Fusidic acid dispensings in older patients was significantly lower among Māori and Pasifika compared with non-Māori/non-Pasifika (both *p* ≤ 0.05, Table 3). Chloramphenicol dispensings in older patients was significantly higher among Pasifika compared with non-Māori/non-Pasifika (*p* = 0.018); there was no significant difference between Māori and non-Māori/non-Pasifika (*p* = 0.057). In children, there were no significant differences in chloramphenicol and fusidic acid dispensings between ethnicities (all *p* ≥ 0.05).

The most socioeconomically deprived patients accounted for the largest proportion of chloramphenicol and ciprofloxacin dispensings (Table 1). This difference was greatest with ciprofloxacin, with quintile 5 accounting for nearly one-third of all dispensings. In contrast, fusidic acid dispensings were more evenly distributed.

Antibiotic dispensing rates were higher in urban health districts. Again, this disparity was most marked with ciprofloxacin dispensings (3.3 ± 0.36 and 1.8 ± 0.24/1000 people/year, in urban and non-urban health districts, respectively). With chloramphenicol dispensings, there were 60.7 ± 5.1 and 51.7 ± 4.9/1000 people/year, in urban and non-urban health districts, respectively. With fusidic acid dispensings, there were 12.6 ± 3.0 and 10.3 ± 2.1/1000 people/year, in urban and non-urban health districts, respectively. The above differences were all statistically significant (all *p* ≤ 0.05).

## 3. Materials and Methods

Ethical approval for this study was obtained from the Auckland Health Research Ethics Committee (reference AH21886). This study analysed deidentified data from a national dispensing dataset of publicly funded pharmaceuticals. Population figures were obtained from the 2006, 2013 and 2018 NZ censuses from Statistics NZ [13]. Population figures for the intervening years were obtained from the previous census.

Every subsidy-eligible community dispensing of the following ocular antibiotics in NZ between 1 January 2010 and 31 December 2019 was included in this analysis: fusidic acid eye drops 1%, ciprofloxacin eye drops 0.3%, and chloramphenicol eye drops 0.5% and eye ointment 1% (both formulations were combined for analysis). These medications cover 99% of all publicly funded ocular antibiotic dispensings in NZ and are eligible for subsidisation when prescribed for approved indications per the NZ Pharmaceutical Schedule; these dispensings are referred to as subsidy-eligible dispensings in this study [1]. In NZ, chloramphenicol can also be dispensed by pharmacists without a prescription; these dispensings are not publicly funded and thus were not captured in the utilised dataset.

Number of dispensings/1000 population was quantified for each year in the study period. This was used to determine mean dispensings/1000 population/year across the study period. Dispensing rates were stratified by patient age and prioritised ethnicity. Dispensing rates by ethnicity were also age-adjusted, using the Māori population as the reference population, to account for the younger age structure of the Māori and Pasifika populations [13].

Dispensings were also analysed by socioeconomic deprivation, based on NZDep2013 Index of Deprivation decile. The deciles were aggregated into equally sized quintiles (with quintile 1 being the least deprived, and quintile 5 the most deprived), and the proportion of dispensings was determined.

Dispensings were also analysed according to the patients’ domicile health district; specifically, health districts were designated urban or non-urban for this analysis. During the study period, NZ was geographically divided into 20 health district offices, which provided and funded public health services within their district. The following health districts were designated urban: Auckland, Waitemata, Counties Manukau, Capital and Coast, Canterbury, Waikato, Bay of Plenty, Southern and Hutt Valley. These served a Major Urban Area (defined by Statistics NZ based on population size)—namely Auckland, Wellington, Christchurch, Hamilton, Tauranga, Dunedin and Lower Hutt [13]. The remaining 11 health districts were designated as non-urban.

The statistical analyses were performed using IBM SPSS Statistics version 26 (Armonk, NY, USA). Statistical analysis was performed using the independent t-test and one-way ANOVA (with post hoc Tukey’s HSD test where applicable). *p* ≤ 0.05 was considered to be statistically significant.

## 4. Discussion

This study examined the utilisation of publicly funded ocular antibiotics over a 10-year period across NZ. Chloramphenicol was by far the most commonly dispensed antibiotic. This differs from antibiotic usage in other countries, with levofloxacin and tobramycin reported to be the most commonly used therapies in China and Spain, respectively [7,8]. Such variations may be attributable to differences in availability, subsidisation and prescribing guidelines between countries [7,8].

Although indications for dispensings were not available in the utilised dataset, high levels of chloramphenicol prescribing in NZ may be partially due to its prophylactic use following cataract surgery, and its empirical use in relatively common ocular conditions such as presumed infective conjunctivitis—particularly in primary care [2,3]. Of note, chloramphenicol is classified as a Restricted medicine in NZ, and is thus available via pharmacist dispensing without a prescription and without subsidisation, as well as by prescription with subsidisation. Pharmacist dispensings were not included in this study, as these were not captured by the utilised dataset and comprehensive data on such dispensings are limited in NZ. A description of such pharmacist dispensings would also be useful, as it has been put forward that the classification of chloramphenicol may have influenced its overall provision in other countries [14,15].

Ciprofloxacin is also commonly used in NZ and remains the first line treatment for microbial keratitis [6]. Due to concerns around the development of resistance, ciprofloxacin is increasingly being reserved for the treatment of bacterial keratitis [6]. Indeed, ciprofloxacin use is tightly controlled and is only subsidised when prescribed for the treatment of bacterial keratitis, as well as a few other infective indications, and the prescription is endorsed accordingly. Some studies have reported increasing ciprofloxacin resistance in NZ, although a large proportion of ocular isolates remain sensitive to ciprofloxacin, as well as to chloramphenicol [9,16,17,18]. In contrast, the proportion of isolates sensitive to fusidic acid may be comparatively smaller (60–74% of staphylococcal ocular isolates) [9].

Overall, antibiotic utilisation was highest in paediatric and geriatric populations, which has also been documented in other countries [8]. In NZ, chloramphenicol dispensing was slightly higher among older patients, which may be partially due to cataract surgery prophylaxis [2,19]. Prolific antibiotic usage in children may be due to the relative prevalence of acute infectious conjunctivitis [20]. However, although antibiotics are often prescribed in bacterial conjunctivitis to speed resolution and thus reduce transmission, there is limited evidence to support this use [21]. In addition, topical antibiotics are also commonly used in ocular adenoviral infections [6]. However, the evidence again does not suggest any benefit, as the risk of secondary bacterial infection is negligible [22].

Ciprofloxacin usage was higher among Māori and Pasifika. It is uncertain whether bacterial ocular infections are also more prevalent among these populations. However, other ocular infections, such as herpes simplex keratitis, have been reported to be more prevalent amongst Māori [23]. Chloramphenicol usage was higher among Pasifika, which may partially be attributed to higher cataract surgery rates [19]. Fusidic acid use was lower among older Māori and Pasifika, which may reflect barriers to healthcare access, and potential undertreatment of more mild external conditions such as blepharitis [24,25].

Antibiotic utilisation was higher in socioeconomically deprived patients, and this was particularly marked with ciprofloxacin. The relationship between the prevalence of ocular infections and socioeconomic deprivation in NZ is unclear. However, systemic antimicrobial consumption in NZ has been linked to socioeconomic deprivation in some studies, which postulated a link between deprivation and higher infection rates [10,12]. On the other hand, antibiotic dispensing was lower in non-urban health districts, again this was particularly marked with ciprofloxacin. This may reflect lower eye care provision in less urbanised areas [26]. Furthermore, urbanity and deprivation-related patterns of antibiotic use may be influenced by other contributing factors. For instance, Māori have a higher proportion of the population residing in less urbanised areas [13]. In addition, higher proportions of Māori and Pasifika reside in areas of higher deprivation [27,28].

This study had some limitations. Firstly, some health districts designated as urban for this analysis (such as the Southern health district) also cover rural areas. Secondly, pharmacist dispensings were not captured by the utilised dataset. Thirdly, indications for dispensings were also not captured by the utilised dataset. This dataset is one of the national administrative health datasets, collected by Manatū Hauora Ministry of Health of the NZ Government. As this is primarily a pharmaceutical dataset for the management of pharmaceutical subsidies, clinical information (such as indication for medicine use) is not collected. However, this analysis leveraged the nationwide coverage of this dataset and the coverage of every subsidised dispensing, to describe population-level patterns in ophthalmic antibiotic consumption. Further research is currently underway, to further describe ophthalmic antibiotic utilisation in NZ, including usage for specific indications.

In summary, the utilisation of publicly funded ocular antibiotics across NZ varies between patient subgroups. Such research will help improve the management and outcomes of related ophthalmic conditions, and support wider initiatives in antibiotic stewardship and access equity.

## Figures and Tables

**Table 1:** Demographics of dispensings included in this analysis.

% of Dispensings		Chloramphenicol	Fusidic Acid	Ciprofloxacin
Age (years)	0–14	34	40	41
	15–39	16	13	21
	40–64	23	21	24
	65 and over	27	26	14
Gender	Male	47	45	51
	Female	53	55	49
Ethnicity	European	67	70	58
	Māori	13	12	20
	Pasifika	8	6	13
	Asian	11	10	8
	Middle Eastern/Latin American/African/Other	1	2	1
Socio-economic position (NZdep13 score quintile)	1 (least deprived)	18	20	18
	2	18	19	16
	3	19	20	17
	4	21	20	18
	5 (most deprived)	24	21	31
Health district	Urban	78	79	85
	Non-urban	22	21	15

**Table 2:** Age-adjusted number of dispensings/1000 population/year, by patient ethnicity.

Age-Adjusted Number of Dispensings/1000 Population/Year (Mean ± SD)	Chloramphenicol	Fusidic Acid	Ciprofloxacin
Māori	53.4 ± 9.3	10.1 ± 3.0	4.2 ± 0.57
Pasifika	65.9 ± 4.1	10.5 ± 3.4	5.3 ± 0.77
Non-Māori/non-Pasifika	57.6 ± 8.6	13.0 ± 3.4	2.8 ± 0.31

**Table 3:** Number of dispensings/1000 population/year, by patient ethnicity and age group.

Number of Dispensings/1000 Population/Year (Mean ± SD)		Chloramphenicol	Fusidic Acid
Māori	0–14 years	88.6 ± 13.4	19.5 ± 6.7
	Over 65 years	93.6 ± 17.6	14.7 ± 2.9
Pasifika	0–14 years	91.0 ± 13.2	17.1 ± 6.1
	Over 65 years	137.5 ± 21.4	17.4 ± 4.6
Non-Māori/non-Pasifika	0–14 years	96.7 ± 7.5	24.6 ± 7.4
	Over 65 years	113.4 ± 15.4	23.2 ± 3.0

## Data Availability

Restrictions apply to the availability of these data from the authors as this is national administrative health data collected and provided by, and used by the authors with the permission of, Manatū Hauora Ministry of Health.
